# Pulmonale Manifestationen bei Long-COVID

**DOI:** 10.1007/s00108-022-01371-3

**Published:** 2022-07-07

**Authors:** Natascha Sommer, Bernd Schmeck

**Affiliations:** 1 Excellence Cluster Cardio-Pulmonary Institute (CPI), Universities of Giessen and Marburg Lung Center (UGMLC), Justus-Liebig-Universität, Klinikstr. 33, 35390 Gießen, Deutschland; 2 Klinik für Innere Medizin mit Schwerpunkt Pneumologie, Schlaf- und Intensivmedizin, Sektion für Atemwegsinfektionen, Philipps-Universität Marburg, Marburg, Deutschland; 3 Institut für Lungenforschung, Universities of Giessen and Marburg Lung Center (UGMLC), Mitglied des Deutschen Zentrums für Lungenforschung (DZL) und des Deutschen Zentrums für Infektionsforschung (DZIF), Philipps-Universität Marburg, Marburg, Deutschland

**Keywords:** Long-COVID/Pathophysiologie, „Severe acute respiratory syndrome coronavirus type 2“ (SARS-CoV-2), Dyspnoe, Husten, Interstitielle Lungenerkrankungen, Long COVID/physiopathology, Severe acute respiratory syndrome coronavirus type 2 (SARS-CoV-2), Dyspnea, Cough, Lung diseases, interstitial

## Abstract

**Hintergrund:**

Pulmonale Manifestationen sind sehr häufige Folgeerscheinungen nach einer Severe-acute-respiratory-syndrome-coronavirus-type-2(SARS-CoV-2)-Infektion, die unter dem Begriff Long-COVID-Syndrom (*COVID* „coronavirus disease“) zusammengefasst werden.

**Ziel und Methoden:**

Zusammenfassung der aktuellen Literatur zu den pulmonalen Manifestationen mit einem Fokus auf Expertenempfehlungen.

**Ergebnisse:**

Dyspnoe ist nach der chronischen Fatigue das häufigste Symptom bei Patienten mit Long-COVID-Syndrom. Auffällige Befunde finden sich vor allem nach schwerem akutem COVID-19-Verlauf und beinhalten radiologische Veränderungen im Sinne interstitieller Lungenerkrankungen, restriktive lungenfunktionelle Befunde und Einschränkungen der Diffusionskapazität als häufigsten pathologischen Befund. Obwohl sich sowohl Beschwerden als auch pathologische pulmonale Befunde im Verlauf bessern, können einige Patienten noch Monate nach der akuten Infektion unter Auffälligkeiten leiden. Dabei ist die Relevanz der pathologischen Befunde sowie eine Beteiligung funktioneller respiratorischer Einschränkungen, einer kardiopulmonalen Dekonditionierung, nichtsomatischer Ursachen und vorbestehender Erkrankungen aktuell unklar. Die diagnostische Abklärung fokussiert entsprechend auf Risikopatienten und schließt neben einer bildgebenden und lungenfunktionellen Abklärung eine Belastungsuntersuchung und bei unklaren Befunden eine Echokardiographie zur Diagnose einer pulmonalvaskulären Komponente ein. Die therapeutischen Möglichkeiten beinhalten aktuell die leitliniengerechte Therapie von Ursachen der Beschwerden (beispielsweise interstitielle Lungenerkrankungen, Husten) und Rehabilitationsmaßnahmen.

**Schlussfolgerung:**

Das aktuelle Wissen zum Krankheitsbild wird ständig erweitert, allerdings existieren aufgrund mangelnder Studienlage noch keine evidenzbasierten Leitlinien zur Diagnostik und Therapie pulmonaler Manifestationen beim Long-COVID-Syndrom.

## Abstract (en)

**Background:**

Pulmonary manifestations are very common sequelae after severe acute respiratory syndrome coronavirus type 2 (SARS-CoV-2) infections, which are summarized under the term long COVID (coronavirus disease) syndrome.

**Aim/methods:**

This article summarizes the current literature on pulmonary manifestations with a focus on expert opinions and recommendations.

**Results:**

After chronic fatigue, dyspnea is the most common symptom in patients with long COVID syndrome. Pathological findings are mainly found after a severe acute course of COVID-19 and include radiological changes with characteristics of interstitial lung diseases, restrictive ventilation patterns and limitations in diffusion capacity as the most common pathological finding. Although both symptoms and pathological pulmonary alterations improve over time, some patients may still suffer from abnormalities months after the acute infection. The relevance of the pathological findings, as well as the involvement of functional respiratory limitations, cardiopulmonary deconditioning, non-somatic causes and pre-existing lung diseases, is currently unclear. The advanced diagnostic assessment thus focusses on high-risk patients and includes, in addition to imaging and pulmonary function tests, a cardiopulmonary exercise test and, if the findings are unclear, an echocardiography to diagnose a pulmonary vascular component. The therapeutic options currently include treatment of the underlying causes of the symptoms (e.g. interstitial lung diseases, cough) according to the respective guidelines and rehabilitation measures.

**Discussion:**

The current knowledge about pulmonary manifestations in long COVID patients is constantly being expanded, but due to limited availability of clinical trials, there are still no evidence-based guidelines for the diagnosis and therapy of pulmonary manifestations in long COVID syndrome.

Obwohl seit Beginn der weltweiten Severe-acute-respiratory-syndrome-coronavirus-type-2(SARS-CoV-2)-Pandemie das Wissen über die „coronavirus disease 2019“ (COVID-19) stark expandiert ist, sind noch viele Fragen bezüglich langfristiger Folgeerscheinungen offen, insbesondere bei aktuellen Virusvarianten. Dieser Beitrag gibt eine aktuelle Übersicht zur Prävalenz, Pathogenese, Diagnose und Therapie pulmonaler Manifestationen des Long- bzw. Post-COVID-Syndroms. Dabei orientiert er sich in wichtigen Punkten an der deutschen S1-Leitlinie [20] und den Expertenempfehlungen der European Respiratory Society [3].

## Pulmonale Symptome bei Long-COVID-Syndrom

### Definition und Häufigkeit

Als Long-COVID-Syndrom werden nach der aktuellen vorläufigen klinischen Falldefinition der Weltgesundheitsorganisation (WHO) verschiedene Beschwerden bezeichnet, die länger als 4 Wochen nach einer SARS-CoV-2-Infektion bestehen bleiben oder nach diesem Zeitraum neu aufgetreten sind und – auch undulierend – über mind. 2 Monate anhalten. Der Long-COVID-Begriff umfasst somit auch das Post-COVID-19-Syndrom, das sich auf Beschwerden ab 12 Wochen nach einer SARS-CoV-2-Infektion bezieht [3, 20, 22].

Aktuell wird geschätzt, dass ungefähr 15 % der COVID-19-Patienten über Wochen bis Monate fortbestehende Symptome nach einer akuten SARS-CoV-2-Infektion aufweisen, wobei die Häufigkeiten in Untersuchungen mit Patienten nach COVID-19-bedingter Hospitalisierung deutlich höher und in bevölkerungsbasierten Umfragen deutlich niedriger liegen können [20, 23]. Risikofaktoren für die Entwicklung eines Long-COVID-Syndroms sind unter anderem höheres Alter und weibliches Geschlecht sowie eine Hospitalisierung und vermehrte Symptome während der COVID-19-Erkrankung, wobei ein direkter Zusammenhang der Häufigkeit des Auftretens mit der Schwere der initialen Erkrankung nicht vollständig gesichert ist und die Erkrankung auch bei milden COVID-19-Verläufen vorkommt [6, 8, 23].

Asthma wurde als Risikofaktor für anhaltende Beschwerden nach COVID-19 identifiziert

Häufigste Symptome des Long-COVID-Syndroms sind Fatigue und Dyspnoe. Inwieweit pulmonale Erkrankungen zur Fatigue beitragen können, ist bisher ungeklärt. Häufig tritt auch Husten über mehrere Wochen und Monate nach COVID-19 auf [22, 23]. Interessanterweise wurde Asthma als Risikofaktor für anhaltende Beschwerden nach COVID-19 identifiziert [6, 8], sodass möglicherweise respiratorische Symptome des Long-COVID-Syndroms auch eine Verschlechterung einer bestehenden Vorerkrankung anzeigen. Auch andere Viruserkrankungen, wie Influenza, „severe acute respiratory syndrome“ (SARS) und „Middle East respiratory syndrome“ (MERS), verursachen langfristige pneumologische und nichtpneumologische Folgeerscheinungen, die Inzidenz scheint aber im Falle von COVID-19 zumindest bei hospitalisierten Patienten im Vergleich zu Influenza erhöht zu sein [1].

### Dyspnoe als Symptom bei Long-COVID

Die Angaben zur Prävalenz von Dyspnoe sind sehr variabel und reichen 1–3 Monate nach einer COVID-19-Erkrankung von 15 % bis 81 % bzw. etwa 1 Jahr nach Erkrankung von 5 % bis 23 % bei ehemals hospitalisierten Patienten. Bei nichthospitalisierten Patienten betragen sie 7 Monate nach der COVID-19-Erkrankung ungefähr 14 % [23]. Die Dyspnoe besserte sich in den Studien bei den meisten Patienten im Verlauf [31, 32, 34], wobei allerdings Untergruppen von Patienten sowohl mit als auch ohne Hospitalisierung identifiziert werden konnten, die noch ein Jahr nach COVID-19-Erkrankung Luftnot angaben [32, 34]. Eine aktuelle Studie aus Wuhan zeigte bei 14 % der Überlebenden 2 Jahre nach einer COVID-19-Erkrankung Luftnot, allerdings wurde in der Prävalenz von Dyspnoe (bestimmt anhand eines Wertes ≥ 1 der „British Medical Research Council (mMRC) dyspnoea scale“ kein Unterschied zur Prävalenz in einer Gruppe passender Nicht-COVID‑19 Erkrankter gefunden [19].

### Husten und thorakale Beschwerden bei Long-COVID

Husten wurde weniger häufig als Dyspnoe berichtet, bei Patienten 1–3 Monate nach der Hospitalisierung waren es 2–42 % [23]. Ein Jahr nach der akuten Erkrankung war die Prävalenz relativ niedrig (2,5 % ehemals hospitalisierter Patienten) und es konnten keine Risikofaktoren für die Persistenz identifiziert werden [12]. Wie bei anderen postinfektiösen Hustensyndromen ist der Husten beim Long-COVID-Syndrom möglicherweise auf eine Hypersensitivität sensorischer Nerven, insbesondere vagaler Nerven zurückzuführen [23]. Thorakale Brustschmerzen wurden in sehr variablen Häufigkeiten angegeben, in den meisten Studien bei bis zu etwa 20 % der Patienten 1–3 Monate nach COVID-19-Erkrankung [8]. Bei ehemals hospitalisierten Patienten nach einem Jahr war die Prävalenz mit 6,5 % ebenfalls niedrig [12].

## Häufige Befunde pulmonaler Manifestationen bei Long-COVID

### Radiologische Veränderungen

Vor allem bei Patienten nach schwerer COVID-19-Erkrankung (mit Intensivaufenthalt, Intubation und gegebenenfalls „acute respiratory distress syndrome“ [ARDS]) werden häufig vielfältige radiologische Veränderungen nachgewiesen, die sich meist im Lauf der Zeit bessern [3, 9, 23]. Zu den prinzipiell reversiblen Auffälligkeiten gehören Milchglastrübungen, die auch typischerweise bei akuter COVID-19-Pneumonie auftreten und meist rückläufig sind. Als wichtigste üblicherweise irreversible Veränderungen treten fibrotische Läsionen wie Retikulationen, Traktionsbronchiektasen und Wabenläsionen auf, die bei 13–27 % der Patienten 3–4 Monate nach einer teilweise sehr schweren COVID-19-Erkrankung beschrieben wurden [23]. Die Ausdehnung der Läsionen ist gering bis mittelschwer und betrifft häufig < 25 % des Lungenparenchyms, wobei unklar ist, ob alle fibroseähnlichen Läsionen irreversible Veränderungen darstellen oder teilweise auch langsam regrediente Infiltrate in Folge einer sich organisierenden Pneumonie [3, 23]. So wurden in einer Studie 12 Monate nach einer COVID-19-Erkrankung mit Hospitalisierung nur bei 1 % der Patienten fibrotische Folgeerscheinungen (im Sinne von „honeycombing“) in der hochauflösenden Computertomographie (HR-CT) gefunden. In den meisten Fällen dieser Studie (66 % der Patienten) wurden nichtfibrotische Veränderungen mit eingeschränkter anatomischer Ausdehnung beobachtet [11]. Ähnliche Befunde ergaben sich im 2‑Jahres-Verlauf nach COVID-19 [19].

### Lungenfunktionelle Befunde

Die radiologischen Veränderungen korrelieren mit leichten lungenfunktionellen Einschränkungen. So zeigt sich lungenfunktionell passend zu einer interstitiellen Lungenerkrankung („interstitial lung disease“ [ILD]) häufig ein restriktives Ventilationsmuster. Allerdings waren die totale Lungenkapazität (TLC) und die Kohlenmonoxiddiffusionskapazität (DLCO) bei Patienten mit fibrotischen Läsionen nur leicht eingeschränkt (TLC 74,1 ± 13,7 % des Soll, DLCO 73,3 ± 17,9 % des Soll; [3, 23]). Auch andere Studien zeigten eine leichte Einschränkung der TLC und DLCO nach mildem bis schwerem COVID-19-Verlauf (TLC 78–102 % des Soll, DLCO 61–81 % des Soll 4 Monate nach einer COVID-19-Erkrankung; [24]). Die lungenfunktionellen Befunde korrelieren mit der Schwere des initialen Verlaufs und bessern sich im Lauf der Zeit, wobei insbesondere die Einschränkung der DLCO über einen längeren Beobachtungszeitraum bis zu einem Jahr persistieren kann [3, 11, 16, 18, 32, 34]. Bei Patienten nach sehr schwerem COVID-19-Verlauf zeigten sich im 2‑Jahres-Verlauf signifikant häufiger Zeichen einer restriktiven Ventilationsstörung und Lungendiffusionsstörung als bei Kontrollpatienten [19]. Die Diffusionsstörung ist damit auch die häufigste Auffälligkeit, die nach einer COVID-19-Erkrankung beobachtet wird.

Lungenfunktionell zeigt sich häufig eine Einschränkung der Diffusionskapazität

Weniger häufig wird eine Einschränkung des Transferkoeffizienten gefunden [3]. Der Transferkoeffizient (KCO) bezieht die DLCO auf das ventilierte Alveolarvolumen (DLCO/VA) und ist im Gegensatz zur DLCO nicht durch eine Einschränkung des ventilierten Alveolarvolumens, etwa bei Adipositas, beeinflusst. Er wird nur durch die Diffusionsstrecke und -fläche der alveolokapillären Membran sowie das kapilläre Blutvolumen bestimmt und ist bei ILD und pulmonalvaskulären Erkrankungen eingeschränkt. Eine kürzlich publizierte Studie unter Einsatz der Magnetresonanztomographie mit hyperpolarisiertem Xenon stützt den Befund einer alveolokapillären Diffusionslimitation bei Patienten mit weitgehend normalem Computertomogramm und lungenfunktionellem Befund [14]. Bei Long-COVID-Patienten mit eingeschränkter DLCO liegt daher wahrscheinlich eine Kombination aus diesen verschiedenen Störungen (weniger ventilierte Alveolen, eingeschränkte Diffusion entlang der alveolokapillären Membran) in individuell unterschiedlichem Ausmaß vor [5].

## Mechanismen und Phänotypen pulmonaler Manifestationen bei Long-COVID

### Mechanismen der Dyspnoe

Die Mechanismen der Dyspnoe nach COVID-19 sind multifaktoriell, es können sich aber bestimmte Phänotypen und mögliche Ursachen abgrenzen lassen, einschließlich direkter Folgen von Lungenparenchymschäden, Atemregulationsstörungen, muskulärer Dekonditionierung und möglicherweise einer kardio- und pulmonalvaskulären Dysfunktion.

### Patienten mit typischer restriktiver Lungenerkrankung vorwiegend nach schwerem COVID-19-Verlauf

Diese Patienten zeigen Zeichen einer interstitiellen Komponente der Lungenerkrankung nach einer schweren COVID-19-Pneumonie. Die Einschränkungen sind am ehesten Folge von direkten virusinduzierten und immunologisch bedingten Organschäden während der akuten Erkrankung. Die Patienten zeigen häufig radiologische Auffälligkeiten sowie typische restriktive lungenfunktionelle Veränderungen mit verminderter TLC und Einschränkung der Diffusionskapazität. Diese Befunde bessern sich im Verlauf der Rekonvaleszenz, wobei die Diffusionskapazität aber auch im Verlauf bis zu einem Jahr nach der COVID-19-Erkrankung noch eingeschränkt sein kann [3, 11, 16, 18, 32, 34].

### Patienten mit persistierender Diffusionseinschränkung

Diese Patienten sind durch fehlende Hinweise auf eine Lungenparenchymbeteiligung bei persistierender Dyspnoe und Leistungseinschränkung charakterisiert. Hier zeigt sich lungenfunktionell und radiologisch ein weitgehend unauffälliger Befund, allerdings können eine Einschränkung der Diffusionskapazität [3, 11, 16, 18, 32, 34] und gegebenenfalls atypische Zeichen einer Restriktion [32] nachgewiesen werden. Diese Patienten sind möglicherweise durch eine Verminderung des alveolär ventilierten Volumens (siehe oben) und/oder auch durch eine persistierende Schädigung der alveolokapillären Membran oder eine pulmonalvaskuläre Erkrankung eingeschränkt.

### Patienten mit möglicher pulmonalvaskulärer Komponente

Aktuell ist die Relevanz einer pulmonalvaskulären Beteiligung beim Post-COVID-19-Syndrom, insbesondere bei initial milder COVID-19-Erkrankung, unklar, da die diagnostischen Nachweismethoden eingeschränkt sind, vor allem im Anfangsstadium bei fehlenden Zeichen einer manifesten pulmonalen Hypertonie (PH). Die generelle Hyperkoagulabilität während einer Hospitalisierung sowie der vaskuläre Tropismus von SARS-CoV‑2 aufgrund der Expression des „angiotensin-converting enzyme 2“ (ACE2) auf endothelialen Zellen begünstigen Thrombembolien, Mikrothromben und eine pulmonalvaskuläre Dysfunktion, die zu einer chronisch thrombembolischen pulmonalen Hypertonie (CTEPH), chronisch thrombembolischen Erkrankung ohne PH (CTED) oder pathologischen Perfusionsumverteilung führen kann [10, 23, 26, 29].

Die Relevanz einer pulmonalvaskulären Beteiligung beim Post-COVID-19-Syndrom ist noch unklar

Bei Patienten nach COVID-19-bedingter Hospitalisierung oder COVID-19-assoziiertem ARDS zeigten sich bis mehrere Monate nach der akuten Erkrankung echokardiographisch Hinweise auf eine PH oder isolierte rechtsventrikuläre Dysfunktion [21, 31] bzw. konnte invasiv eine PH bei symptomatischen Patienten nachgewiesen werden [28]. Allerdings sprach bei Patienten nach milder COVID-19-Erkrankung eine unauffällige pulmonale Hämodynamik unter Ruhebedingungen und unter Belastung gegen eine relevante PH [30]. Ob bei diesen Patienten eine funktionelle Perfusionsumverteilung ohne PH eine Rolle in der Entstehung der Long-COVID-Symptomatik spielt, ist aktuell unklar.

### Patienten mit Atemregulationsstörungen und muskulären Ursachen

Spiroergometrisch gibt es Hinweise, dass die Belastungseinschränkung bei manchen Patienten vorwiegend durch ein gestörtes Atemmuster („dysfunktionale Atmung“), eine kardiopulmonale Dekonditionierung oder eine periphere muskuläre Einschränkung verursacht sein kann [23, 30]. Myopathien wurden nach SARS-CoV-2-Infektion auch im Vergleich zu Influenzainfektionen vermehrt diagnostiziert [1]. Invasive hämodynamische Messungen unter Belastung ergaben, dass die Belastungseinschränkung bei Post-COVID-19-Patienten mit mildem COVID-19-Akutverlauf eher durch eine periphere als durch eine zentrale kardiopulmonale Limitation verursacht wird mit einer begleitenden hyperventilatorischen Antwort [30]. Zusätzlich zeigen sich auch isolierte Atemregulationsstörungen oder eine Schwäche der inspiratorischen Atemmuskulatur bei Post-COVID-19 [23]. Aktuell ist unklar, ob bei der dysfunktionalen Atmung nach COVID-19-Erkrankung psychische Faktoren eine Rolle spielen oder eine zerebrale Atemdysregulation [23].

### Patienten mit obstruktiver Lungenerkrankung

Obstruktive Muster und eine bronchiale Hyperreagibilität, wie sie bei anderen Infektionen postinfektiös häufig beschrieben wurde, waren eher selten zu beobachten [3, 24, 32]. Die Rolle einer „small airway disease“ und Bronchiolitis obliterans ist aktuell unklar, radiologisch konnte aber ein „air trapping“ bei einem hohen Prozentsatz von Patienten auch nach mildem COVID-19-Verlauf nachgewiesen werden, wobei dieses mit lungenfunktionellen Zeichen der Überblähung korrelierte [7].

## Pathophysiologische Grundlagen

**Figure 1:**
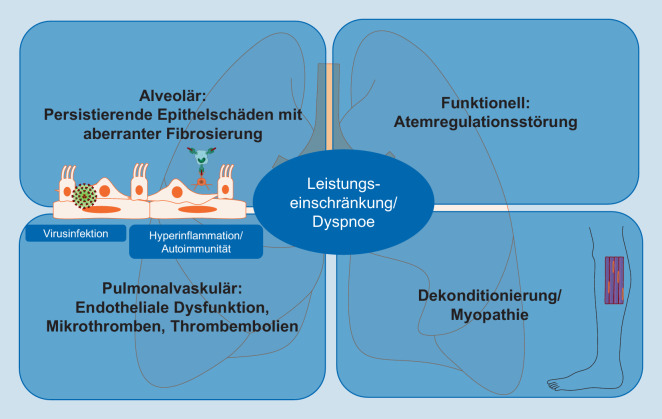

Als Ursache für die kardiopulmonalen Einschränkungen beim Long-COVID-Syndrom kommen folgende Ursachen in Betracht, die möglicherweise bei unterschiedlichen Phänotypen der Erkrankung in unterschiedlichem Ausmaß zur Pathogenese beitragen (siehe auch Abb. 1):

- Persistierende Organschäden, die durch in der Akutphase ausgelöste virusspezifische oder immunologisch bedingte Prozesse verursacht werden, beispielsweise
  - ILD oder
  - Folgen einer akuten Lungenembolie
- Persistierende Hyperinflammation und Autoimmunphänomene, die chronische Lungengewebsumbauprozesse, eine Gefäßdysfunktion, (Kardio‑)Myopathien, Hyperkoagulabilität und eine neurogene Beteiligung begünstigen können [2, 8, 23, 27]
- Unspezifische Mechanismen, wie sie auch bei anderen Viruserkrankungen auftreten, beispielsweise postinfektiöser Husten
- Dekonditionierung mit oder ohne dysfunktionelle Atmung/Atemmuskelschwäche
- Verschlechterungen vorbestehender – gegebenenfalls bisher nicht diagnostizierter – Erkrankungen
- Nichtsomatische Ursachen

Unklar ist, inwiefern eine Viruspersistenz in peripheren Organen zum Long-COVID-Syndrom beitragen kann [2, 17, 20, 23]. Es ist wichtig zu beachten, dass replikationsfähige Viren selten länger als 20 Tage nach Auftreten der Symptome nachgewiesen werden konnten [33]. Allerdings kann nicht ausgeschlossen werden, dass das Virus an für das Immunsystem schlecht zugänglichen Orten persistiert. Inwiefern eine Impfung die Bekämpfung von Restviren oder eine Restitution der immunologischen Homöostase unterstützen kann oder das Risiko eines Auftretens von COVID-19-Folgeerscheinungen nach Durchbruchinfektionen senkt, wird in einem weiteren Beitrag dieses Schwerpunkts diskutiert.

## Diagnostische Empfehlungen

**Figure 2:**
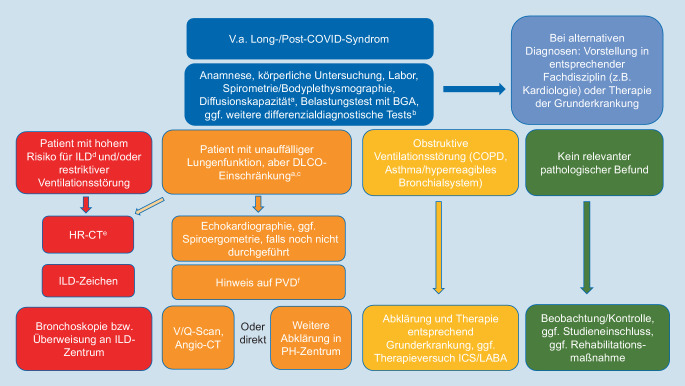

Verschiedene Fachgesellschaften und Expertengremien haben im Laufe der Pandemie jeweils adaptierte Empfehlungen zur Diagnostik abgegeben, wobei die meisten eine Abklärung von Patienten empfehlen, die länger als 3 Monate nach der Infektion symptomatisch sind, zudem werden gegebenenfalls vorzeitige Untersuchungen von Patienten nach schwerer COVID-19-Erkrankung angeraten [3, 8, 17, 20]. Die neuesten Empfehlungen und relevante zusätzliche Expertenmeinungen, die sich auf Patienten ab 3 Monate nach COVID-19-Erkrankung beziehen, sind in Abb. 2 integriert und werden im Weiteren aufgeführt.

### Primärärztliche Versorgung

In der primärärztlichen Versorgung wird bei persistierenden Beschwerden nach einer COVID-19-Erkrankung eine Basisdiagnostik mit ausführlicher Anamnese und Labordiagnostik empfohlen, bei Patienten ohne Warnhinweise auf eine schwerwiegende Erkrankung ein abwartendes Vorgehen [20]. Bei Patienten mit stark beeinträchtigenden Symptomen (insbesondere nach einem schweren Verlauf der akuten Erkrankung), Verschlechterung der Symptomatik oder sonstigen Unklarheiten sollte eine erweiterte Diagnostik erfolgen.

### Erweiterte pneumologische Diagnostik

Wie in Tab. 1 aufgeführt, können verschiedene pulmonale, kardiovaskuläre und muskuläre Erkrankungen sowie eine Dekonditionierung und nichtsomatische Problematiken zu Dyspnoe, Husten und thorakalen Beschwerden beim Long-COVID-Syndrom beitragen, wobei zur Abklärung bestimmte Prädispositionen und Befundkonstellationen wegweisend sein können.

**Table 1:** 

Differenzialdiagnosen		Anamnese	Diagnostik^a^	Spiroergometrische Veränderungen^b^
Pulmonal	Restriktive Lungenerkrankung (interstitielle Lungenerkrankung)	Risikofaktoren: hohes Alter, schwere COVID-19-Erkrankung (z. B. mit Beatmung, Infiltrationen, erniedrigter T‑Zell-Zahl, Erhöhung von LDH, IL‑6 und D‑Dimeren), Zustand nach ARDS [3]	TLC, VC, DLCO; ggf. Röntgenuntersuchung des Thorax; HR-CT bzw. Low-dose-CT	Restriktives Atemmuster (hohe Atemfrequenz bei niedrigem Tidalvolumen); eingeschränkter Gasaustausch mit Abnahme des arteriellen Sauerstoffpartialdrucks unter Belastung^c^
	Obstruktive Lungenerkrankung (chronisch-obstruktive Lungenerkrankung, Asthma, Bronchiolitis obliterans)	Pneumologische Anamnese	FEV_1_/VC, RV, PEF; ggf. Provokation (medikamentös oder Belastung); bei Verdacht auf eine postinfektiöse Bronchiolitis obliterans: CT in Exspiration („air trapping“)	Obstruktives Atemmuster, dynamische Überblähung; ggf. eingeschränkter Gasaustausch bei Emphysem
	Diffusionsstörung (interstitielle Lungenerkrankung, pulmonalvaskuläre Erkrankung)	Risikofaktoren: Zustand nach Lungenembolie/VTE, erhöhtes Risiko für VTE (z. B. gemäß IMPROVE-Score, D‑Dimer-Erhöhung)	DLCO; DLCO/VA oder DLNO (spezifischer für Veränderungen des Gasaustauschs entlang der alveolokapillären Membran, siehe „Lungenfunktionelle Befunde“); ggf. Ventilations‑/Perfusionsszintigraphie und/oder Angio-CT	Eingeschränkter Gasaustausch mit Abnahme des arteriellen Sauerstoffpartialdrucks unter Belastung^c^; bei pulmonalvaskulärer Erkrankung: Abnahme des Partialdrucks des endtidalen Kohlendioxids unter Belastung; bei Rechtsherzinsuffizienz: vorzeitige Abflachung des Sauerstoffpulses
	Atemmechanische Einschränkung (inspiratorische Atemmuskelschwäche, dysfunktionale Atmung)	Ggf. Nijmegen-Fragebogen für dysfunktionale Atmung	Ggf. Atemmuskelfunktionsmessung, ggf. Hyperventilationstest	Restriktives Atemmuster ohne Gasaustauschstörung, bei Hyperventilation: erhöhtes Atemäquivalent für Kohlendioxid
Kardial	Myokarditis, Kardiomyopathie	Kardiologische Anamnese	Elektrokardiogramm, Echokardiographie, ggf. Kardio-MRT (nur bei therapeutischer Relevanz^d^), ggf. weitere kardiologische Diagnostik	Bei Linksherzinsuffizienz: vorzeitige Abflachung des Sauerstoffpulses
Peripher	Myopathie, Perfusionsdysregulation	Muskelschmerzen	Normalbefunde, angiologische Diagnostik	Eingeschränkte periphere Sauerstoffaufnahme (invasiv mit Messung der gemischtvenösen Sättigung)
Dekonditionierung	–	Trainingsstatus vor und nach COVID-19-Erkrankung	Normalbefunde	Niedrige anaerobe Schwelle und überschießende Herzfrequenzsteigerung bei niedriger Belastung; im Übrigen unauffällige Befunde
Nichtsomatisch	–	Psychosoziale Belastung, ggf. entsprechende Scores	Normalbefunde	Normalbefund

*ARDS* „acute respiratory distress syndrome“, *COVID* „coronavirus disease“, *CT *Computertomographie, *DLCO* Kohlenmonoxiddiffusionskapazität, *DLCO/VA* Transferkoeffizient, *FEV*_*1*_ forcierte exspiratorische Einsekundenkapazität, *HR-CT* hochauflösende Computertomographie, *IL‑6* Interleukin‑6, *IMPROVE* International Medical Prevention Registry on Venous Thromboembolism, *LDH* Laktatdehydrogenase, *MRT* Magnetresonanztomographie, *PEF* „peak expiratory flow“, *RV* Residualvolumen, *TLC* totale Lungenkapazität, *VA* ventiliertes Alveolarvolumen, *VC* Vitalkapazität, *VTE* venöse Thrombembolien

^a^Laborparameter: Aktuell sind keine spezifischen Laborparameter zur Diagnose oder Verlaufskontrolle einer Long-COVID-Erkrankung etabliert. Organspezifische Blutparameter sollten gemäß der entsprechenden Verdachtsdiagnose bestimmt werden

^b^Andere Belastungstests: Als Screeningtest auf eine Belastungshypoxämie kann bei Patienten mit einer Sauerstoffsättigung von über 96 % in Ruhe, bei denen eine Spiroergometrie nicht durchgeführt werden kann, gegebenenfalls auch eine Belastung mittels Geh- oder Aufstehtest durchgeführt werden. Ein Abfall der arteriellen Sauerstoffsättigung > 3 % bedarf einer weiteren Abklärung

^c^Eine Normalisierung des Gasaustauschs unter Belastung deutet auf eine Verbesserung des Perfusions-Ventilations-Verhältnisses hin, beispielsweise durch Rekrutierung von in Ruhe minderbelüfteten Lungenbereichen (etwa bei Adipositas)

^d^Die Prävalenz kardiologischer Folgeerscheinungen im Sinne einer Myokarditis und Kardiomyopathie ist sehr variabel und die Relevanz unklar. Eine Kardio-MRT wird nur in Ausnahmefällen empfohlen [27]

### Spezifisches Vorgehen bei verschiedenen Patientengruppen

Einen Überblick zur erweiterten diagnostischen Abklärung der Dyspnoe gibt Abb. 2.

#### Patienten nach schwerem COVID-19-Verlauf, die ein hohes Risiko für eine interstitielle Lungenerkrankung oder Zeichen einer restriktiven Ventilationsstörung aufweisen

Diese Patienten sollten eine bildgebende Abklärung erhalten und gegebenenfalls in einem ILD-Zentrum vorgestellt werden. Generell sollte eine HR-CT gegenüber einer Röntgenaufnahme des Thorax oder Ultraschalluntersuchung der Lunge bevorzugt werden, bei jungen Patienten kann auch eine Low-dose-CT durchgeführt werden [3].

#### Patienten mit unauffälliger Bildgebung (oder Symptomatik unverhältnismäßig zur Bildgebung) und unauffälligen dynamischen/statischen Lungenparametern im Lungenfunktionstest

Diese Patienten sollten eine weitere Abklärung einer pulmonalvaskulären Erkrankung mittels Echokardiographie und Angio-CT bzw. zum Ausschluss einer CTEPH/CTED eine Ventilations‑/Perfusions(V/Q)-Szintigraphie erhalten und gegebenenfalls in einem PH-Zentrum vorgestellt werden [3, 13]. Insbesondere die Wertigkeit der V/Q-Szintigraphie bei einem peripheren Phänotyp der pulmonalvaskulären Erkrankung bei COVID-19 wurde bereits früh hervorgehoben [10]. Die genaue Sequenz der Untersuchungen und die Indikation für die Bildgebung sind aktuell unklar, können sich aber an der Abklärung einer CTEPH orientieren, in deren Algorithmus vor Durchführung eines obligaten V/Q-Scans ein echokardiographisches Screening steht [13]. Anhand des echokardiographischen Befunds und unter Abwägung anderer Hinweise auf eine CTEPH (z. B. durchgemachte Lungenembolien, Diffusionsstörungen und spiroergometrische Befunde) wird über das weitere Vorgehen entschieden [13]. Dieses Vorgehen wurde auch für Post-COVID-19-Patienten vorgeschlagen [10].

Offene Frage bleibt, welche Patienten mit eingeschränkter DLCO eine weitere Abklärung mittels HR-CT und/oder Echokardiographie erhalten sollten. Da aktuell unklar ist, wie häufig eine therapierelevante pulmonalvaskuläre Erkrankung bei symptomatischen Long-COVID-Patienten auftritt, kann die Frage nicht abschließend beantwortet werden. Außerdem ist unklar, zu welchem Zeitpunkt eine erweiterte Diagnostik durchgeführt werden sollte, da sowohl Symptome, als auch DLCO-Auffälligkeiten im Verlauf häufig rückläufig erscheinen. Mögliche Entscheidungshilfen für die weitere Abklärung bieten das individuelle Risikoprofil des Patienten für die Entwicklung einer interstitiellen oder pulmonalvaskulären Erkrankung (insbesondere einer CTEPH), die Schwere der DLCO (bzw. DLCO/VA) -Einschränkung und mögliche alternative Ursachen dafür. In jedem Fall sollte bei auffälligen Befunden eine Verlaufskontrolle erfolgen, da sich Folgeerkrankungen, insbesondere die CTEPH, erst im langfristigen Verlauf nach einer „Honeymoon-Periode“ in fortgeschrittenem Stadium symptomatisch manifestieren können [23].

#### Patienten mit obstruktivem lungenfunktionellem Befund und/oder anamnestischem Hinweis auf Asthma oder ein hyperreagibles Bronchialsystem

Bei diesen Patienten sollte gegebenenfalls eine Provokationsuntersuchung und Asthmaabklärung durchgeführt werden. Das Vorgehen zur Abklärung einer Bronchiolitis obliterans ist aktuell nicht geklärt.

#### Patienten ohne auffälligen pulmonalen Befund oder Hinweis auf pulmonalvaskuläre Erkrankung

Bei diesen Patienten sollte gegebenenfalls eine weitergehende Abklärung mittels Atemmuskeltestung oder Test auf eine hyperventilatorische Antwort durchgeführt werden. Des Weiteren sollte gegebenenfalls eine Vorstellung bei einem Kardiologen oder die Abklärung einer psychosomatischen Erkrankung erfolgen.

#### Patienten mit Husten und unspezifischen thorakalen Beschwerden

Die Abklärung von Husten sollte entsprechen der aktuellen Leitlinie zur Abklärung von akutem, subakutem und chronischem Husten erfolgen. Subakuter Husten (bis 8 Wochen nach akuter Infektion) ist ein häufiges unspezifisches Symptom nach einer viralen Atemwegsinfektion und kann meist abwartend beobachtet werden (außer bei Auftreten von „red flags“). Die Abklärung von thorakalem Druck sollte nach allgemeinen Prinzipien erfolgen; es sollten muskuloskeletale und unspezifische Beschwerden von schwerwiegenden kardiovaskulären Komplikationen unterschieden werden.

## Therapeutische Ansatzpunkte

### Etablierte Therapien

Zielgerichtete medikamentöse Therapieoptionen bei Long-COVID existieren bisher nicht. Die deutsche S1-Leitlinie empfiehlt eine leitliniengerechte symptomatische Behandlung gemäß pathologischen Befunden, etwa bei postinfektiösem Husten. Bei Post-COVID-19-Patienten mit bronchialer Hyperreagibilität führte die Therapie mit Beclometason/Formoterol (100 µg/6 µg 2‑0‑2 für 6–8 Wochen) zu Symptomfreiheit innerhalb von 3 Monaten [24]. Bei Verdacht auf eine interstitielle bzw. pulmonalvaskuläre Erkrankung sollte die Vorstellung in einem entsprechenden Zentrum und gegebenenfalls die Einleitung einer Therapie erfolgen. Bei Patienten mit organisierender Pneumonie war die Therapie mit Cortison in einer Beobachtungsstudie wirksam [25].

Einen speziellen Stellenwert in der Therapie des Long-COVID-Syndroms nimmt die Physiotherapie und Rehabilitation ein. Ein weiterer Beitrag in diesem Schwerpunkt beschreibt Details zu Rehabilitationsmaßnahmen bei Long-COVID.

### Prophylaktische Antikoagulation zur Prävention pulmonalvaskulärer Erkrankungen

Eine generelle Thrombembolieprophylaxe nach einer COVID-19-Erkrankung wird aktuell nicht empfohlen. Denn trotz erhöhten Risikos von venösen Thrombembolien (VTE) nach einer COVID-19-Erkrankung (geschätzt mit einer Inzidenz von 2,5 % der Patienten nach Hospitalisierung [17]) wird das Risiko einer Thrombembolie im Vergleich zu möglichen Blutungskomplikationen anhand der aktuellen Datenlage als eher gering angesehen [15]. Allerdings kann eine Antikoagulation bei Patienten mit einem geringen Blutungsrisiko und einem hohen VTE-Risiko in Betracht gezogen werden [15]. Diese Empfehlung wird durch die MICHELLE-Studie unterstützt, die eine signifikante Reduktion von VTE und Todesereignissen nach Entlassung ergab, wenn Patienten mit erhöhtem VTE-Risiko mit 10 mg/Tag Rivaroxaban prophylaktisch antikoaguliert wurden [15].

### Neue Therapieansätze in klinischen Studien

Aktuell wird eine Vielzahl an klinischen Interventionsstudien zum Long-COVID-Syndrom durchgeführt, teilweise mit unklarer Begründung bezüglich eines spezifischen Long-COVID-Krankheitsmechanismus [4]. Viele der Studien betreffen nichtpharmakologische Interventionen, die respiratorische Symptome adressieren, neben allgemeinen Trainingsprogrammen auch Atemphysiotherapie. Zu den pharmakologischen Interventionen bezüglich respiratorischer Folgeerscheinungen zählen unter anderem die Behandlung mit Montelukast (NCT04695704) und Deupirfenidon (NCT04652518). Antiinflammatorische Substanzen werden zur Therapie verschiedener Symptome des Post-COVID-19-Syndroms untersucht. Ein Beispiel ist Leronlimab, ein monoklonaler Antikörper gegen den CC-Chemokin-Rezeptor 5 (NCT04678830).

## Fazit für die Praxis

- Dyspnoe und Husten sind häufige persistierende Symptome nach einer COVID-19-Erkrankung.
- Die funktionellen Auswirkungen von Lungenparenchymschäden sind im Allgemeinen begrenzt und die Dyspnoe bessert sich im Laufe der Zeit bei den meisten Patienten, auch wenn eine Untergruppe von Patienten bis zu mind. einem Jahr nach COVID-19 eine anhaltende Dyspnoe aufweist.
- Die Mechanismen der Dyspnoe nach COVID-19 sind multifaktoriell, einschließlich einer Lungenparenchymbeteiligung (v. a. bei initial schwerem Verlauf), Atemregulationsstörungen, kardiovaskulärer/pulmonalvaskulärer Dysfunktion, muskulärer Dekonditionierung und nicht-somatischer Ursachen.
- Sowohl bisher unentdeckte als auch aggravierte vorbestehende pulmonale Erkrankungen können zum Beschwerdebild beitragen.
- Die Ursachen und Therapieoptionen bei Dyspnoe und Belastungseinschränkung in der Patientengruppe mit Long-COVID müssen weiter untersucht werden – auch interdisziplinär.
